# Neurological impairment among heterozygote women for X-linked Adrenoleukodystrophy: a case control study on a clinical, neurophysiological and biochemical characteristics

**DOI:** 10.1186/1750-1172-9-6

**Published:** 2014-01-13

**Authors:** Clarissa Troller Habekost, Pedro Schestatsky, Vitor Felix Torres, Daniella Moura de Coelho, Carmen Regla Vargas, Vitor Torrez, Jean Pierre Oses, Luis Valmor Portela, Fernanda dos Santos Pereira, Ursula Matte, Laura Bannach Jardim

**Affiliations:** 1 Post-Graduation Program in Genetics and Molecular Biology, Universidade Federal do Rio Grande do Sul (UFRGS), Porto Alegre, Brazil; 2 Post-Graduation Program in Biochemistry, Universidade Federal do Rio Grande do Sul (UFRGS), Porto Alegre, Brazil; 3 Department of Biochemistry, Universidade Federal do Rio Grande do Sul (UFRGS), Porto Alegre, Brazil; 4 Department of Analysis, Universidade Federal do Rio Grande do Sul (UFRGS), Porto Alegre, Brazil; 5 Department of Internal Medicine, Universidade Federal do Rio Grande do Sul (UFRGS), Porto Alegre, Brazil; 6 Medical Genetics Service, Hospital de Clínicas de Porto Alegre, Porto Alegre, Rio Grande do Sul, Brazil; 7 Neurology Service, Hospital de Clínicas de Porto Alegre, Porto Alegre, Rio Grande do Sul, Brazil; 8 Laboratory of Genetic Identification, Hospital de Clínicas de Porto Alegre, Porto Alegre, Rio Grande do Sul, Brazil; 9 Laboratory of Gene Therapy, Hospital de Clínicas de Porto Alegre, Porto Alegre, Rio Grande do Sul, Brazil; 10 Laboratório de Neurociências Clínicas, Centro de Ciências da Vida e da Saúde, Universidade Católica de Pelotas, Pelotas, Brazil; 11 Instituto Nacional de Genética Médica Populacional (INAGEMP), Porto Alegre, Brazil; 12 Instituto Nacional de Ciência e Tecnologia em Excitotoxicidade e Neuroproteção (INCTEN), Porto Alegre, Brazil

**Keywords:** X-linked adrenoleukodystrophy, X-ALD heterozygote females, X-ALD carriers, JOA, SSPROM, Neuron-specific enolase, Evoked potentials, Nerve conduction, X inactivation, Spastic paraplegia

## Abstract

**Background:**

Neurologic impairments in female heterozygotes for X-linked Adrenoleukodystrophy (X-ALD) are poorly understood. Our aims were to describe the neurological and neurophysiological manifestations of a cohort of X-ALD heterozygotes, and to correlate them with age, disease duration, mutations, X-inactivation and serum concentrations of a marker of neuronal damage, neuron-specific enolase (NSE).

**Methods:**

All 45 heterozygotes identified in our region, with previous VLCFA and molecular diagnosis, were invited to be evaluated through myelopathy scales JOA and SSPROM, nerve conduction studies and somatosensory evoked responses. X inactivation pattern was tested by HUMARA methylation assay. Serum NSE was measured by eletrochemiluminescense.

**Results:**

Thirty three heterozygote women were recruited: 29 (87%) were symptomatic. Symptomatic and asymptomatic women presented different m ± sd ages (43.9 ± 10.2 versus 24.3 ± 4.6), JOA (14.5 ± 1.7 versus 16.6 ± 0.2) and SSPROM (86.6 ± 7.9 versus 98.4 ± 1.1) scores (p < 0.05). Both JOA (r = −0.68) and SSPROM (r = −0.65) correlated with age, irrespectively of the disease status (p = 0.0001, Spearman). Delayed latencies in the central ascending conduction studies on the lower limbs were present in 72% of all heterozygotes, and correlated with SSPROM (r = −0.47, p = 0.018, Spearman). NSE values were higher in heterozygote than in control women (12.9 ± 7 and 7.2 ± 7 ng/ml, p = 0.012, Mann-Whitney U). Mutation severity and inactivation patterns were not associated with neurologic status.

**Conclusion:**

Neurologic manifestations, clearly related to age, were quite common in the present cohort. JOA and SSPROM scales were able to discriminate the asymptomatic from the symptomatic heterozygotes. Both scales might be useful tools to follow disease progression, in future studies.

## Background

X-linked adrenoleukodystrophy (X-ALD, OMIM #300100) is the most common peroxisomal disorder worldwide [1,2]. Affecting the metabolism of saturated very long chain fatty acids (VLCFAs), X-ALD is caused by a defect in *ABCD1* gene that codes for ALD protein [3], a peroxisomal membrane protein that belongs to the ATP-binding cassette superfamily of membrane transport proteins [4]. *ABCD1* mutations give rise to obvious phenotypes in hemizygote men, such as the classical cerebral form of ALD (CALD) and the adrenomyeloneuropathy (AMN), among others [4].

A very limited number of studies addressed manifestations in female carriers; some authors stated that affected women are under recognized and undertreated [5]. Early reports said that 20 to 50% of heterozygotes might present a mild to severe myeloneuropathy that resembles AMN, this clinical picture being related to ageing [6]. The myelopathic component of AMN is characterized by a distal axonopathy that affects the dorsal column and the corticospinal tract [7]. Neurophysiologic studies showed central and peripheral abnormalities and suggested the prominence of axonal dysfunction [8].

VLCFA were established a long time ago as biomarkers for X-ALD. Elevated plasma VLCFA levels are the diagnostic gold-standard for X-ALD in men and present 15-20% of false negative results in women, for whom a molecular investigation is the main diagnostic tool [4].

In addition to diagnostic concerns, there are no reasonable mechanistic explanations for the variability regarding the presence and absence of neurological impairments in heterozygote females. Although brain VLCFA levels correlated with CALD in hemizygotes [9], plasma VLCFA levels have never been related to X-ALD phenotypes in men and women [4]. It is well known that genotypes do not correlate with phenotypes, in X-ALD [4]. Skewed X chromosome inactivation was also investigated, bringing contradictory results [10-12]. Alterations in oxidative stress parameters such as thiobarbituric acid-reactive substances [13] have been described in female carriers, without further exploring their potential associations with phenotypes.

Therefore, clinical and biochemical biomarkers of disease progression in heterozygotes for X-ALD are lacking. In this sense, we aimed to describe the symptomatic status of our X-ALD heterozygote cohort; to measure the neurological manifestations through myelopathy scales JOA and SSPROM, peripheral nerve conduction studies and somatosensory evoked responses; to measure plasma levels of a neuron disease marker, neuron-specific enolase (NSE); and to look for associations between these parameters and independent variables such as age, age at onset, mutations, X inactivation pattern and plasma VLCFA.

## Methods

### Population and clinical evaluations

All women previously identified as heterozygotes for X-ALD, in South Brazil, were invited to participate in this study. All had molecular analyses and VLCFA dosages performed in our institution, as reported elsewhere [14]. To be included, they had to be over 18 years old and give the informed consent. Exclusion criteria were the presence of any abnormality on the following exams at recruitment: lymphocytes count, hemoglobin, erythrocytes median corpuscular volume, sedimentation rate, vitamin B12, thyroid stimulating hormone, proteinogram, VDRL (Venereal disease research laboratory), FTA-Abs (Fluorescent Treponemal Antibody- absorption), antibodies anti-HTLV (Human T lymphotropic virus) and Anti-HIV (Human Immunodeficiency Virus), oxalacetic and glutamic pyruvic transaminases, qualitative urine test, thrombocytes, glucose and creatine.

The interview started with an open question about any disability or presence of symptoms in the heterozygote. After that, the interviewer (CTH) described the symptoms of spasticity, paraparesis, sensory losses and loss of sphincter control, and explained that these symptoms could be present in heterozygote females. After that, the interviewer asked again about the presence and age at onset (AO) of motor disability, sensory losses and/or loss of sphincter control and the answer to this was taken into account. Heterozygote women were then classified according to their responses; as symptomatic if complaints of neuropathic pain, paresthesia, sphincter dysfunction or paresis were present, or as asymptomatic if these complaints were absent. Data such as age, AO, disease duration (DD), family and ABCD1 mutations were collected.

Neurological impairment and disability were evaluated through two myelopathy scales: the Japanese Orthopaedic Association (JOA) [15] and the Severity Score System for Progressive Myelopathy (SSPROM) [16]. JOA includes questions about motor disability of upper and lower limbs, about sensory losses and about sphincter function. SSPROM includes questions about these same domains, plus questions about motor strength and tonus/reflexes. In this sense, both scales measure disability as well as neurological impairment.

We elected two SSPROM domains to measure disability. SSPROM’s motor disability domain was based on and mirrors the Overall Disability Sum Score [17], while SSPROM’s sphincter domain was based and recapitulates the “sphincter dysfunction” domain of Kurtzke functional systems scores [18]. An overall disability evaluation was then measured by these two SSPROM domains (0 to 50 points; Table 1). Since a minimally important difference has not been determined for the disability parameters so far, we have arbitrated that women with an overall score ≤ 47 points were already disabled.

**Table 1:** **Clinical characteristics of the local cohort of X**-**ALD heterozygote women**

	**X**-**ALD heterozygotes**			p*
	**All heterozygotes participant**	**Symtomatic**	**Asymtomatic**	
N (families)	(16) 33	29	4	
Age at examination	41.2 ± 11.9	43.9 ± 10.2	24.3 ± 4.6	0.005
		(24–61)	(20–30)	
Age at onset of symptoms		39.4 ± 10 (21–59)		
Disease duration		4.5 ± 3.3		
JOA (range: -2 to17)	14.8 ± 1.8	14.5 ± 1.7	16.6 ± 0.2	0.005
SSPROM (range: 0 to 100)	88 ± 8.4	86.6 ± 7.9	98.4 ± 1.1	0.001
SSPROM Motor Disability (range: 0 to 30)	27.6 ± 2.6	27.3 ± 2.6	30 ± 0	0.023
SSPROM Motor Strength (0 to 20)	18.9 ± 2	19.1 ± 1.3	17.5 ± 5	ns
SSPROM sensory losses (0 to 20)	16.4 ± 2.4	16.1 ± 2.3	18.5 ± 9	ns
SSPROM spasticity/ hyperreflexia (0 to 10)	6.1 ± 2.1	5.7 ± 1.8	9.4 ± 1.2	0.05
SSPROM sphincter contro (0 to 30)	27.6 ± 2.6	17.8 ± 2.3	20 ± 0	ns

*Mann-Whitney U.

In order to produce a “disease severity score” where the neurological impairment was corrected by the disease duration, the SSPROM reciprocate of each woman was divided by disease duration. This severity score was then compared among groups stratified by risk factors such as mutation position, mutation severity, NSE and inactivation patterns.

Blood samples were taken and neurophysiological studies (described below) were performed within 15 days of evaluation.

### Neurophysiological studies

Motor and sensory nerve conduction studies in several nerves were performed in the right side of the body (Table 2). Motor conduction velocity (m-NCV), distal latency and amplitude for both compound muscle action potential (CMAP) and sensory nerve action potential (SNAP) were measured according to standard techniques in which normal values were considered within 2-SD from the mean [19].

**Table 2:** **Nerve conduction studies according to the presence or absence of symptoms of 22 ×**-**ALD heterozygote women**

**Neuroconduction studies, right side (Mean ±** **sd) (range)**		**All 22 heterozygotes**	**Presence of symptoms**		p*
			**Symptomatic (20)**	**Asymptomatic (2)**	
Motor conduction velocity (m/s)	Median	57 ± 6.2 (47–73)	56.9 ± 6.5	58.9 ± 2.2	ns
	(results above the cutoff value of 49,2)		(2)	(0)	
	Fibular	48.1 ± 5 (40–55)	47.3 ± 4.9	53.2 ± 1	ns
	(results above the cutoff value of 45.3)		(3)	(0)	
	Tibial	49.5 ± 4.6 (44–63)	49.2 ± 4.6	54.4	ns
	(results above the cutoff value of 39.9)		(0)	(0)	
Sensitive conduction velocity (m/s)	Median	49.4 ± 3.9 (42–59)	49.2 ± 4	52 ± 2	ns
	(results above the cutoff value of 51.7)		(12)	(0)	
	Ulnar	52.6 ± 6 (44–66)	52.8 ± 6.3	51.3 ± 0.6	ns
	(results above the cutoff value of 53.8)		(3)	(0)	
	Fibular superficial	53.7 ± 5.7 (44–64)	53.1 ± 5.4	58.4 ± 7.5	ns
	(results above the cutoff value of 35)		(0)	(0)	
Motor conduction amplitude (mV)	Median	9 ± 3.6 (2–16)	9 ± 3.7	9.2 ± 0.5	ns
	(results under the cutoff value of 3.3)		(1)	(0)	
	Fibular	4 ± 1.9 (1–7)	4 ± 1.8	3.9 ± 3.2	ns
	(results under the cutoff value of 1.6)		(1)	(0)	
	Tibial	7.7 ± 4.7 (2 to 19)	7.6 ± 5	8.1 ± 2.3	ns
	(results under the cutoff value of 2,8)		(1)	(0)	
Sensitive conduction amplitude (μV)	Median	14.5 ± 5.8 (5 to 29)	14.5 ± 5.9	15.2 ± 7	ns
	(results under the cutoff value of 15.1)		(3)	(0)	
	Ulnar	11.3 ± 6 (5 to 27)	11.4 ± 6.4	10.9 ± 3.3	ns
	(results under the cutoff value of 5.8)		(0)	(1)	
	Fibular	15.3 ± 6.6 (5 a 30)	15.3 ± 6.5	15.4 ± 11	ns
	(results under the cutoff value of 10)		(2)	(1)	

*Mann-Whitney U.

Somatosensory evoked responses (SSER) were measured using the 10/20 electrode placement international system and recorded according to standard techniques [19]. The latencies of N8, P40 and N50 were recorded after tibial nerve stimulation, and N9 and N20 after median nerve stimulation. Only P40 and N20 latencies were included into regression analyses. Normal latency values were considered according to Chiappa [20] and based on the patient’s height. The upper limits of normal values were given in Table 3.

**Table 3:** **Somatosensory evoked responses according to the presence or absence of symptoms of 25 ×**-**ALD heterozygote women**

**Somatosensory evoked responses**		**All 25 heterozygote females**	**Presence of symptoms**		**p**
			**Symptomatic women: 23**	**Asymtomatic women: 2**	
Upper Limbs (N20, or somatosensory cortex contralateral to the wrist stimulated) in ms	Mean ± sd	19.2 ± 1.1	20.1 ± 4.2	18 ± 0.9	ns *
	Above the cutoff value of 20.5 ms	6/25	6/23	0/2	ns #
Lower Limbs (P40, or somatosensory cortex ipsilateral to the tibial nerve stimulated) in ms	Mean ± sd	38 ± 2,5	55.3 ± 32.8	38.9 ± 3.8	ns *
	Above the cutoff value of 41,5 ms	18/25	17/23	1/2	ns #

* Mann-Whitney U; m/s = meters per second; mV – milivolts; μV = microvolts.

Both, nerve conduction studies and SSER were performed using a Nihon –Kohden (*Neuropack S1* MEB-9400 K) 4 channel EMG/EP system.

### VLCFA

Plasma docosanoic (C22:0), tetracosanoic (C24:0) and hexacosanoic (C26:0) acids were obtained previously to the present study, and were analyzed as described by others [21]. Values of C26:0 (in µm/L) and of discriminant factor 3,805(C24:0/C22:0) + 5,296(C26:0/C22:0) + 5,15(C26:0) [22] were studied in the present heterozygote females. The discriminant factor’s value of 10.86 discriminated X-ALD heterozygote from normal homozygote women, with 15% of false-negative results.

### Enolase quantification

Serum NSE was measured using an eletrochemiluminescent assay provided by Roche DiagnosticsR, Indianapolis, IN, a double sandwich assay that uses an antibody anti-NSE bound with ruthenium (luminescent label). The reaction and quantification were performed by Elecsys-2010 (Roche). No hemolyzed samples were used. The assay was carried out in duplicate and the confidence variance was within 5%. NSE concentrations were also measured in thirteen unrelated, normal women used as a control group.

### Inactivation studies

Genomic DNA was obtained from peripheral leukocytes by the salting out procedure [23]. X-inactivation patterns were assessed for skewing with the human androgen-receptor locus (HUMARA) methylation assay [24]. Prior to digestion, informative women were identified by PCR at HUMARA locus and capillary electrophoresis. For the informative women (n = 20), digestion of 100 ng genomic DNA with the methylation-sensitive restriction endonuclease, *Hpa*II, and subsequent PCR amplification of the HUMARA polymorphic CAG repeat was used to determine X-inactivation status in X-ALD heterozygotes. The degree of chromosome X inactivation was calculated using the formula [(d1/u1) / (d1/u1 + d2/u2)], where d = digested, u = undigested sample, and the two allele peaks (1 and 2) are compared [25]. Inactivation ratios less than 70:30 were considered random. Ratios greater than or equal to 70:30 and less than or equal to 90:10 were considered moderately skewed. Ratios greater than 90:10 were considered highly skewed [26].

### Statistical analyses

Patient characteristics are given as mean ± SD and range, when applicable. Categorical variables were represented by absolute and relative frequencies and were compared through Fisher exact test. The majority of continuous variables did not show a normal distribution on the Shapiro-Wilk test, and therefore were tested by Mann-Whitney U, Spearman correlation and, when applicable, by stepwise linear regression. Statistical significance was defined as p < 0.05. All statistical tests were performed in PASW 18.3.

## Results

### Clinical findings

Forty five heterozygote women (belonging to 25 families) had been detected by molecular studies in our institution, from 2008 to 2013. Thirty three heterozygotes (or 73% of the total heterozygotes detected in our region) gave their consent and were included in this study. The another 27% (12 women) were lost: six were not found by two phone calls and letter, two refused to participate in the study and another four fulfilled exclusion criteria. The mean ± sd ages of the 33 participants and of the 12 non-participants, in 2013, were of 41.2 ± 11.9 and 40.9 ± 15, respectively (ns, t test). Twenty-nine of the 33 participants classified themselves as symptomatic and four, as asymptomatic, after learning about X-ALD symptoms in females. Their general characteristics are present in Table 1.

Symptomatic were significantly older and presented worse JOA and SSPROM scores and higher disability scores than asymptomatic women. Significant differences in SSPROM scores were due to a high proportion of symptomatic females (27/29) with spasticity/hyperreflexia; and to motor disabilities (Table 1). The auto-classification and the disability scores did not perfectly match: normal scores in the overall disability evaluation were obtained in all four asymptomatic but also in 15/29 symptomatic women. Overall disability was related to SSPROM and JOA scores, but not to age, age at onset of symptoms, neurophysiologic studies and other biomarkers (data not shown).

**Figure 1:**
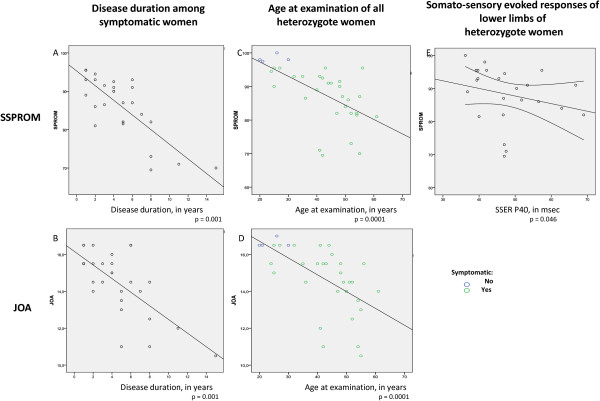
**Associations relating JOA and SSPROM scales to independent variables age, disease duration and somato**-**sensory evoked responses. (A and B)** Disease duration of symptomatic heterozygotes. **(C and D)** Age at examination all heterozygotes. **(E)** Somato-sensory evoked responses of lower limbs of all heterozygotes.

Among symptomatic women, although SSPROM correlated with age and DD, only DD was maintained on linear regression (r = −0.73, p = 0.0001, Spearman; B = −1.91, EP = 0.28). When all heterozygote women were analyzed together, the association with age was confirmed (r = −0.65, p = 0.0001, Spearman; B = −0.43, EP = 0,10, by Linear regression) (Figures 1A and 1C).

Similar associations were found regarding to JOA. Among symptomatic women, although JOA scores were at first correlated with age, AO and DD, only DD was significantly related to JOA on stepwise linear regression (r = − 0.61, p < 0.001, Spearman; B = −0.37; EP = 0.07). When all heterozygotes were analyzed together, JOA correlated with age (r = −0.68, p = 0.0001, Spearman; B = −0.09; EP = 0.02, Linear regression) (Figures 1B and 1D).

Mean ± sd of disease severity, or 1/(SSPROM x DD), among symptomatic women, were 3.7 ± 2.6. This score did not correlate with age or any other parameters.

Symptomatic classification produced more associations with independent parameters than the disability classification, in this cohort. Due to that, it was maintained as the stratification criteria between the present heterozygote women, when necessary.

### Nerve conduction studies

Twenty-two out of 33 heterozygotes (20 symptomatic and 2 asymptomatic women) performed neurophysiologic studies. The others denied for personal reasons. Nineteen (including one asymptomatic heterozygote) presented at least one altered proof. Results are presented in Table 2.

All m-NCV and most of s-NCV were normal, and there were no correlations between NCVs and symptomatic status, age, AO, DD, disability status, JOA, SSPROM, disease severity, inactivation studies or plasma NSE concentrations (data not shown). Twelve out of 20 symptomatic heterozygotes showed reduced s-NCV of the median nerve, measured on the elbow. This finding was unrelated to any variable under study (data not shown).

No evidence of generalized axonal damage was found in any of the women studied, since the majority of CMAPs and SNAPs amplitudes were between the normal range. The amplitude of fibular superficial nerve SNAPs correlated with AO among the heterozygotes (r = 0.55, p = 0.015, Spearman).

### Somatosensory evoked responses

Twenty-five heterozygote females (including those 22 females with nerve studies) were tested for SSE. Eighteen (17 symptomatic) presented delayed latencies in the central ascending conduction studies starting on the lower limbs (evoked potential P40) (Table 3). Latencies of the evoked potentials were not related to age, disability, disease severity or to the symptomatic status of the heterozygote women (ns, Spearman and Mann-Whitney U tests). SSPROM correlated weakly with lower limbs evoked potential latencies (r = −0.47, p = 0.018, Spearman, Figure 1E).

### Mutations found in *ABCD1* gene

The mutations detected on *ABCD1* gene are depicted on Additional file 1: Table S1. These mutations were stratified according to their exonic position in the gene as well as divided according to their severity in missense or nonsense/frameshift. Mutation severity or position did not correlate either to age at onset of symptoms or to disease severity (Mann-Whitney U and Kruskal-Wallis, data not shown).

### X Chromosome inactivation patterns

Twenty heterozygotes were informative in the technique applied to determine the inactivation pattern. Six had a skewed inactivation pattern of 70:30 or more. Skewed inactivation was not associated to symptomatic state (ns, Fisher exact test), disability, SSPROM, JOA, disease severity (ns, Spearman) or AO (ns, Linear Regression). A curious trend to relate larger skewing with age was also seen (r = 0.37, p = 0.1, Spearman).

### NSE

**Figure 2:**
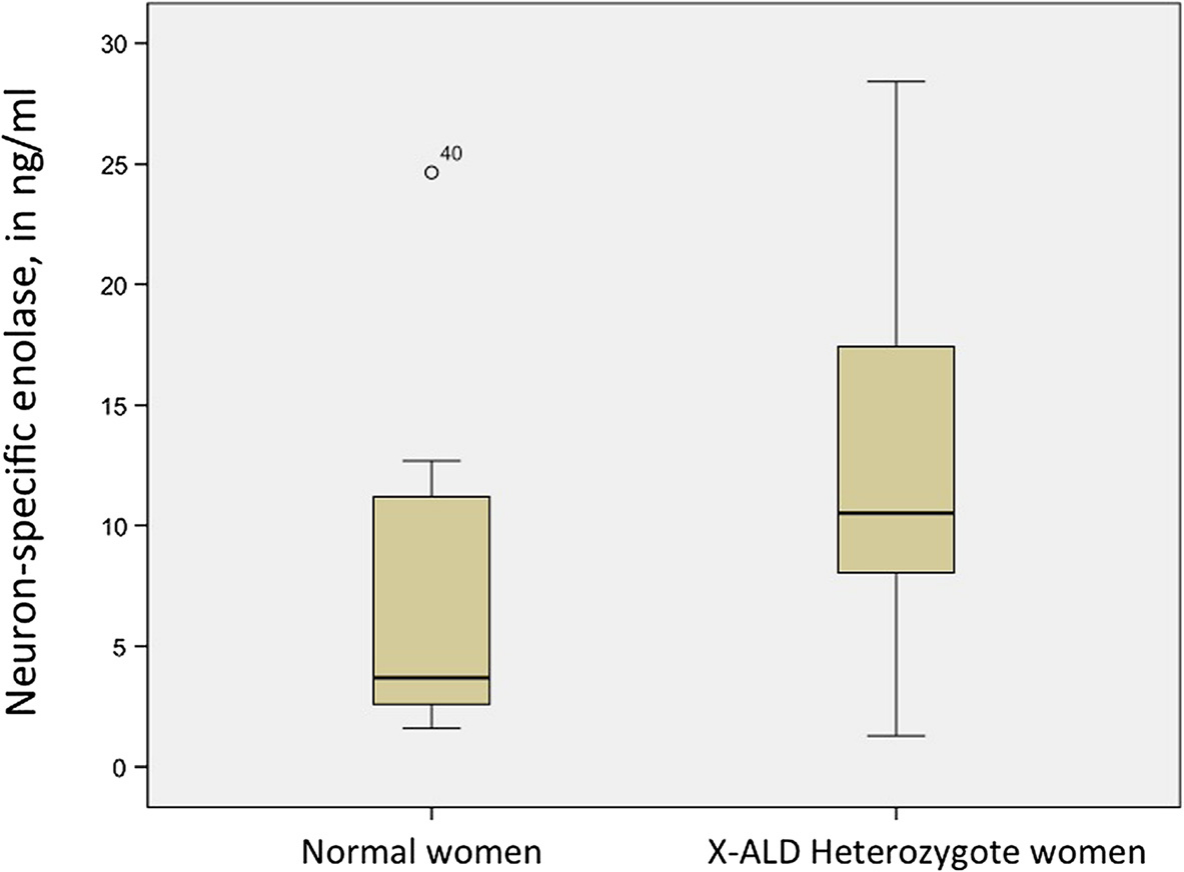
**NSE among the X**-**ALD heterozygotes and the control women.**

The medium ± sd values of NSE were higher in heterozygotes than in controls (12.9 ± 7 versus 7.2 ± 7 ng/ml, p = 0.012, Mann-Whitney U, Figure 2). Both heterozygote and control groups had similar ages (41.2 ± 11.9 and 35.5 ± 10.4 years, ns, M-W U).

NSE was not correlated with age, either among all women or just among heterozygotes. No correlations were found between NSE and AO, disability or symptomatic status, inactivation studies, position of mutation in ABCD1 gene, SSPROM, JOA, disease severity, evoked responses or other neurophysiology parameters (data not shown).

### VLCFA

VLCFA had been determined at the time of diagnosis of the present 33 heterozygotes, sometimes several years before the present evaluation. Their mean ± sd ages at the collection were of 30 ± 11.3 (range: 7 to 43) years-old. In the case of symptomatic women, for whom the symptom onset has been determined as year zero, blood collections were done at a −3.8 ± 4.5 (range: -12 to 3) years from the start of their symptoms. Correlations of VLCFA levels with age at the time of blood collection and with time from disease start were then tested. There were direct correlations between age and both C26:0 and VLCFA discriminant factor (r = 0.58, p = 0.011 and r = 0.60, p = 0.007, Spearman, Additional file 2: Figure S1).

## Discussion

We examined 73% of all known heterozygote women for X-ALD followed in our institution, and detected significant clinical and neurophysiologic impairments in 87% of them. The clinical scales JOA and SSPROM were able to differentiate symptomatic from asymptomatic heterozygotes. Both scales were associated with DD of the symptomatic cases and with age in the overall group. Abnormalities in somatosensory evoked responses, mostly from the lower limbs, were quite frequent. A plasma protein related to neuronal damage, NSE, was associated with the heterozygote state. All these might be candidates as biomarkers for disease progression in heterozygotes for X-ALD.

Quantifying physical disability and neurologic burden is essential to understanding the progression rate of any disease [5]. In the present report, three levels of consequences related to the heterozygote state for X-ALD were measured: subjective perception, neurologic impairment and disability. Being symptomatic is a yes/or/no subjective perception. Neurologic impairment was measured by JOA and SSPROM scales, and by neurophysiologic studies. Disability reflects dysfunctions on performance and activity; we measured it by using specific domains of SSPROM. Disability needs an arbitrary cutoff. Disability scores obtained in the present group were very mild. Due to that, we decided to include even small disabilities in the dichotomic, positive group. We have then stratified heterozygote women both by their symptomatic and disability states.

We have obtained 29 symptomatic and 4 asymptomatic women; in contrast, there were 14 disabled and 19 non-disabled women. Although the disability stratification seemed to produce more even groups, it failed to be associated with age or with DD. In contrast, symptomatic status correlated with age, JOA, and SSPROM; moreover, DD - in other words, the duration of symptoms - correlated with both JOA and SSPROM. Therefore, symptomatic classification produced more useful information than its counterpart (disability).

Neurological impairment was better evaluated by clinical scales than by neurophysiology. Both JOA and SSPROM scales were able to discriminate symptomatic from asymptomatic heterozygotes, with almost no overlap, correlated very well with DD in the symptomatic group, and with age in the overall group.

The lack of any substantial alterations on peripheral neurophysiology reported here corroborates the findings of Schmidt and Cols [8]. One interesting finding was the direct correlation of fibular SNAP with AO. Reduced SNAP is commonly related to an inherent susceptibility of the fibular nerve for entrapments. Why did it correlate with AO, is an issue that deserves investigation in other heterozygotes.

After clinical scores, the SSER of lower limbs have shown the most prevalent abnormalities in heterozygote women. Similar results have been seen by others [8,27-30] SSER of lower limbs correlated with SSPROM and this might be taken as an external validation for SSPROM usefulness among X-ALD heterozygotes. On the other hand, SSER were not associated with any other measurement of disease progression like age and disease duration.

X chromosome inactivation has been studied as a modifying factor that could influence clinical presentation in women with X linked disorders. Relating a skewed inactivation in peripheral leukocytes to a neuronal disorder, as in the heterozygote women with X-ALD, is a complex hypothesis. If this association would exist, at least two possible mechanisms should be invoked: a good mirroring between neurons and leukocytes, where a skewed X inactivation in neurons would be related to differences in clinical presentation; and/or an intercellular mechanism, operating from skewed cells other than neurons. Differently from some authors [12] but similar to others [11], we did not find any association between inactivation and symptomatic status or neurological scores. We observed a trend relating an increased skewing with age, which is in accordance with literature [31].

VLCFA also increased with age, at least in this cohort, whose blood collections had been done between 7 and 43 years of age. Other authors have observed a similar finding in this age group, with decreasing VLCFA concentrations in women older than 50 years old [32]. Although both VLFCA and skewing inactivation increased with age in our group, no association was found with increasing neurological symptoms.

NSE is a cytoplasmatic glycolytic enzyme found in neurons and cells with neuroendocrine differentiation. Since NSE is not physiologically secreted, an increase of its serum concentrations can be associated with structural damage to neuronal cells [33]. NSE concentrations were higher in heterozygote women than in controls, in the present study. Since NSE was not associated with symptomatic or disability status, nor with any variable related to disease progression, we were not able to show a clear potential for NSE as a biomarker for disease progression in this disease.

According to former studies, around 50% of X-ALD heterozygotes showed some degree of neurologic involvement [6]. Our results were quite different: 87% were symptomatic, with neurological impairments as measured by JOA and SSPROM scores. Our large proportion of symptomatic women was quite surprising. Although one cannot rule out a selection bias favoring the recruitment of symptomatic heterozygotes, the similitude between the mean ages of the recruited and of the non-recruited heterozygote women works against this conjecture. Our rates might be explained by different measurements of neurological burden: perhaps former reports relied on disabilities rather than impairments. Our experience showed that disabilities were mild in general, with the exception of sphincter dysfunctions (Table 1). Impairments, on the other hand, were clearly present, the major impact being the corticospinal involvement (Table 1).

Emotional issues should also be remembered. Male X-ALD is a catastrophic disease for families. A mother or a female caregiver of an affected boy usually lives in very stressful conditions, where priorities are put in hemizygote men rather than in her well-being. Maybe the actual numbers of symptomatic heterozygote females had been previously underestimated, due to a hasty report of these women about their healthy states. The hypothesis that the majority of heterozygote females will present a neurologic impairment is not negligible. Moreover, the associations between JOA and SSPROM with age might suggest that the symptomatic status is a matter of time and that, as heterozygote females grow older, they get symptomatic.

With the prominent involvement of pyramidal tracts, X-ALD presenting in symptomatic heterozygotes and as male AMN, can be seen as one form of complicated spastic paraplegia (SPG) [34]. Therapeutic trials in men and women with AMN and in SPGs in general are expected in a near future, and it will be important to decide what scales to use on them. Recently, some studies have measured the neurological impairment of SPGs by the Spastic Paraplegia Rating Scale (SPRS) [35]. In contrast, we have used two instruments fitted to measure both disability and impairment, scales that might be called disease severity scoring systems (or 3S): the scales JOA and SSPROM. SSPROM was developed to follow metabolic progressive myelopathies such as AMN; so far, only the present and the description studies have used it [16]. JOA was developed to follow cervical compression myelopathies [15], and it is probably the most used neurologic scale for myelopathies so far. For a clinical trial, several properties of the chosen scale will be essential to guarantee the best design, such as validity, reliability and sensitivity to change. At this very moment, it is not clear what scale will be best fitted to a future trial for AMN. Comparisons of these characteristics among different scales are needed and should be done in a near future.

## Conclusions

Neurologic impairments are frequent and are clearly related to age and to the CNS involvement, in heterozygote females. SSER of lower limbs and plasma NSE concentrations were altered in the majority of heterozygote females. Since they did not differentiate between symptomatic and asymptomatic heterozygotes, nor were related to age or disease duration, we cannot postulate them as good candidates as biomarkers of disease progression. VLFCA and skewing inactivation were also unable to separate symptomatic from asymptomatic heterozygotes. Both increased with age, but dissociated from the increasing neurological symptoms. Among the number of potential biomarkers of disease progression, JOA and SSPROM presented the most robust associations with disease duration and age. Besides that, SSPROM correlated with SSER of lower limbs. We suggest that JOA and SSPROM might be valuable instruments in future natural history studies in heterozygotes for X-ALD.

## Abbreviations

AMN: Adrenomyeloneuropathy; AO: Age at onset; C22:0: Docosanoic acid; C24:0: Tetracosanoic acid; C26:0: Hexacosanoic acid; CALD: Cerebral form of ALD; CMAP: Compound muscle action potential; DD: Disease duration; HUMARA: Human androgen-receptor locus; JOA: Japanese Orthopaedic Association scale of myelopathy; m-NCV: Motor conduction velocity; NSE: Neuron-specific enolase; SNAP: Sensory nerve action potential; SPG: Spastic paraplegia; SPRS: Spastic Paraplegia Rating Scale; SSER: Somatosensory evoked responses; SSPROM: Severity Score System for Progressive Myelopathy; VLCFA: Very long chain fatty acids; X-ALD: X-linked Adrenoleukodystrophy.

## Competing interests

The authors declare that they have no competing interests.

## Authors’ contributions

CTH conceived the study, participated in the design of the study, carried out the recruitment, interviews and clinical studies, and helped to draft the manuscript. PS carried out the SSER studies and participated in the analysis. PS and VTF carried out peripheral neurophysiology studies and participated in the analysis of the data. DMC performed VLCFA analyses and helped in the coordination of data. CRV performed VLCFA analyses and helped in the acquisition of funding. VT performed NSE analyses and participated in the analysis of data. LVP performed NSE studies and helped in the acquisition of funding. FSP carried out the molecular genetic studies and participated in the analysis. UM participated in the molecular analysis and in the coordination of data. LBJ conceived the study, participated in the design and coordination of the study, performed the statistical analysis, and drafted the manuscript. All authors read and approved the final manuscript.

## Supplementary Material

Additional file 1: Table S1 Mutations found at ABCD1 gene in the present X-ALD heterozygote women, and the distribution of ages at examination and at onset of symptoms. Click here for file

Additional file 2: Figure S1 Associations between age and (6A) C26:00 plasma levels and (6B) VLCFA discriminant factor 3,805(C24:0/C22:0) + 5,296(C26:0/C22:0) + 5,15(C26:0). Click here for file
