# Supplementary material for: Neurological impairment among heterozygote women for X-linked Adrenoleukodystrophy: a case control study on a clinical, neurophysiological and biochemical characteristics
Source: Orphanet J Rare Dis. 2014 Jan 13;9:6. doi: 10.1186/1750-1172-9-6 (PMC3896743; doi:10.1186/1750-1172-9-6)
Supplement: Additional file 1: Table S1 — Mutations found at ABCD1 gene in the present X-ALD heterozygote women, and the distribution of ages at examination and at onset of symptoms. [file 1750-1172-9-6-S1.pdf]

Supplemental Table 1 – Mutations found at ABCD1 gene in the present X-ALD heterozygote women, and the distribution of ages at examination and at onset of symptoms.

Mutations (exon)	Number of women<br><br>Total (asymptomatic women, when present)	Age at examination<br><br>median ± dp (variation), when possible	Age at onset of symptoms
	33<br>(4)	41.2 ± 11.9	39.4 ± 10<br>(21-59)
p.Ala232fsX64,<br>family 21<br>(Exon 2)	1	24	22
p.Gly266Arg,<br>family 12<br>(Exon 2)	2	25 and 47	21 and 42
p.Tyr296Cys,<br>family 24<br>(Exon 2)	2	43 and 44	38 and 42
p.Trp326X,<br>family 6<br>(Exon 2)	2	25 and 50	24 and 43
p.Ser358X,<br>family 37<br>(Exon 2)	1	36	33
p.Arg401Trp,<br>family 32<br>(Exon 3)	1	48	45
p.Arg401Gly,<br>family 52<br>(Exon 3)	1	35	34
p.Arg401Trp,<br>family 58<br>(Exon 3)	1	40	39
p.Gly512Ser,<br>family 1<br>(Exon 6)	2<br>(1)	52	46
p.Arg518Gln,<br>family 10<br>(Exon 6)	7	44.6 ± 8<br>(32-55)	36.7 ± 7<br>(26-44)
p.Glu577X,<br>family 8<br>(Exon 7)	2<br>(1)	55	50
p.Trp601X,<br>family 3<br>(Exon 8)	1<br>(1)		?
p.Ser606Leu,<br>family 2<br>(Exon 8)	2<br>(1)	49	43
p.Arg617His,<br>family 4<br>(Exon 8)	5	48 ± 12<br>(27-56)	45 ± 11<br>(26-55)
p.Leu628Glu,	1	45	41

family 27<br>(Exon 9)			
p.Trp679X,<br>family 23<br>(Exon 10)	2	41 and 61	39 and 59
