# Supplementary figures and images for: Neurological impairment among heterozygote women for X-linked Adrenoleukodystrophy: a case control study on a clinical, neurophysiological and biochemical characteristics
Source: Orphanet J Rare Dis. 2014 Jan 13;9:6. doi: 10.1186/1750-1172-9-6 (PMC3896743; doi:10.1186/1750-1172-9-6)

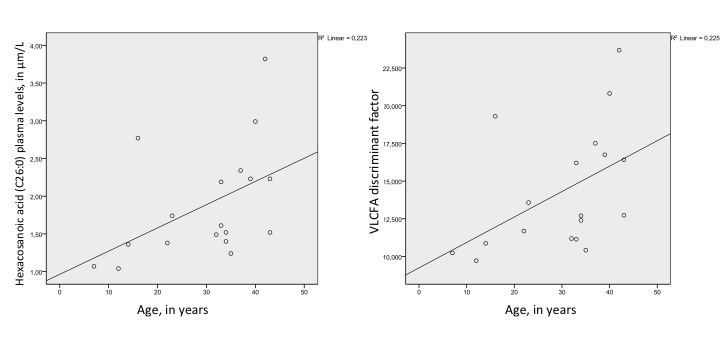

Supplement: Additional file 2: Figure S1 — Associations between age and (6A) C26:00 plasma levels and (6B) VLCFA discriminant factor 3,805(C24:0/C22:0) + 5,296(C26:0/C22:0) + 5,15(C26:0). [file 1750-1172-9-6-S2.JPG]
